# *In vivo* evaluation of the anti-obesity effects of combinations of *Monascus* pigment derivatives†

**DOI:** 10.1039/c9ra08036h

**Published:** 2020-01-08

**Authors:** Deokyeong Choe, Hyun Ho Jung, Daehwan Kim, Chul Soo Shin, Tony Vaughn Johnston, Seockmo Ku

**Affiliations:** Fermentation Science Program, School of Agriculture, College of Basic and Applied Sciences, Middle Tennessee State University Murfreesboro TN 37132 USA seockmo.ku@mtsu.edu; Department of Biotechnology, College of Life Science and Biotechnology, Yonsei University Seoul 03722 South Korea; Department of Biology, Hood College Frederick MD 21701 USA

## Abstract

The prevention and treatment of obesity using naturally derived compounds is desirable in terms of marketing and safety in the nutraceutical and functional food markets. One of the noticeable effects of *Monascus* pigment derivatives is the inhibition/deactivation of lipid metabolism. Our earlier studies reported that threonine (Thr), tryptophan (Trp), and 2-(*p*-tolyl)-ethylamine (TEA) derivatives of *Monascus* pigment showed cholesterol-lowering, lipase-inhibitory, and adipogenic differentiation-inhibitory activities, respectively. In this work, we investigated the *in vivo* anti-obesity effects of a combination of Thr, Trp and TEA derivatives. C57BL/6 mice were fed a high-fat diet (HFD) and simultaneously administered one of three 1 : 1 mixtures of Thr, Trp, and TEA derivatives. After 10 weeks of feeding, the weight gains of mice fed with three combined derivatives decreased by 20.3–37.9%, compared to mice fed the HFD. The epididymal adipose tissue (EAT) weights of mice fed with the combined derivatives decreased by 42.3–60.5% compared to the HFD group, and their EAT size decreased. Transverse micro-CT imaging revealed reduction of the subcutaneous and visceral fat layers of test mice. Our results confirm that *Monascus*-fermented pigment derivatives have *in vivo* anti-obesity effects and their combinations provide a higher efficacy in the reduction of body weight and EAT weights as well as lipid accumulation in mice. The key to accomplishing high anti-obesity effect was combining Thr and Trp derivatives, which provide higher effectiveness than other combined derivatives. These observations offer a potential use of *Monascus* pigment derivatives as a therapeutic approach to prevention and/or treatment of obesity.

## Abstract 2

The combination of threonine and tryptophan derivatives of *Monascus* pigment showed the high anti-obesity effect in mice.

## Graphical abstract

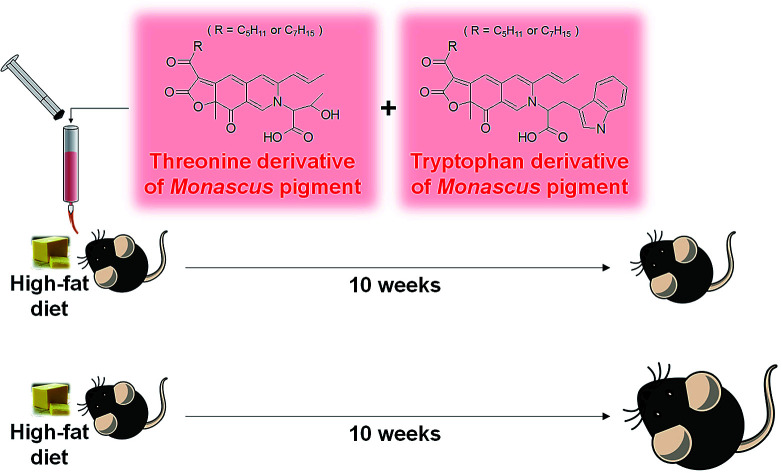

## Introduction

Obesity, a disease characterized by excessive fat accumulation, has rapidly increased worldwide and is reaching pandemic levels.^1^ Being overweight and obese are major factors that substantially increase the risk of diseases, including type-2 diabetes mellitus, hypertension, coronary artery disease, fatty liver, stroke, dementia, myocardial infarction, osteoarthritis, and several cancers (kidney, breast, liver, colon, and endometrial cancers).^2–4^

According to the Milken Institute,^5^ obesity costs the U.S. healthcare system approximately $480.7 billion annually. A recent study estimated that the global obesity treatment market will be US $2.49 billion by 2020.^6^ Several anti-obesity drugs (*e.g.* phentermine, fluoxetine, sibutramine, orlistat and rimonabant) and surgical treatments (Roux-en-Y gastric bypass, adjustable gastric band, sleeve gastrectomy, and biliopancreatic diversion) have been developed, but obesity patients and the general public have limited access to them due to cost, efficacy and safety issues.^7,8^

With these concerns and the demand for cost-effective treatments and drugs, bioactive secondary metabolites synthesized by microorganisms and/or herbs have received attention from casual consumers as alternative strategies for the treatment of obesity.^9^ Among the various naturally derived biogenic compounds that have anti-obesity effects, multiple pigments, including anthocyanins,^10^ carotenoids,^11^ chlorophyll,^12^ and *Monascus* sp. pigments,^13^ have received attention from academia due to their functionality and applicability to foods.

A variety of research groups and scholars have investigated these pigments as alternatives for the treatment of obesity in safe and efficient ways. Naturally occurring colored pigments have long been utilized as coloring agents for foods, cosmetics, textiles and alcoholic beverages in Asia, indicating a low safety risk.

*Monascus* sp. pigments are perhaps the best-known microbial colorants, and are produced by *Monascus* species fungal fermentation.^14^*Monascus* sp. pigments comprise six major azaphilone pigment compounds which range in color from red (rubropunctamine and monascorubramine) to yellow (monascin and ankaflavin) and orange (rubropunctatin and monascorubrin).^15,16^ The production of *Monascus* metabolites and their colors changes depending on *Monascus* growth, culture conditions and morphological changes in the cells.^17–20^ For example, trans-etherification of octanoic acid contributes to the generation of orange pigments while the reduction of orange pigments causes the formation of yellow and red pigments.^19,21^ Of these three colors, it is known that orange *Monascus* pigments can be transformed into derivatives by aminophilic reaction.^22^ Specifically, orange *Monascus* pigments are transformed to red pigments by replacing the pyranyl oxygen with a primary amine, such as a peptide, protein, amino sugar, amino alcohol, chitosan, or nucleic acid.^19,23^

In recent years, various derivatives of orange *Monascus* pigments have been produced with amino acids and amine compounds by Shin's group.^24–32^ These derivatives have been shown to exhibit multiple *in vitro* functional activities, including anti-microbial,^24^ anti-viral,^29^ and anti-melanogenesis effects.^31^ Among the various derivatives created thus far, there are several reports on *Monascus* pigments exhibiting biological activities related to lipid metabolism. The tryptophan (Trp) and 2-(*p*-tolyl)-ethylamine (TEA) derivative of *Monascus* pigment have been shown to be a potential inhibitor for lipase^25^ and adipogenic cell differentiation,^28^ respectively. The threonine (Thr) derivative has also been observed to inhibit 3-hydroxy-3-methyglutaryl coenzyme A (HMG-CoA) reductase in cholesterol-synthesizing metabolism.^26^ Since the Trp, TEA, and Thr derivatives exhibit lipid-related activities by different mechanisms, their combination has the potential to provide enhanced or synergistic anti-obesity effects. However, to our best knowledge, no study has been conducted to evaluate the effect of combined *Monascus* pigment derivatives on anti-obesity.

The main objective of this study was to evaluate the anti-obesity effects of combined *Monascus* pigment derivatives. Derivatives of *Monascus* pigments were produced by *Monascus* cultivation and three 1 : 1 mixtures of derivatives were prepared. Both control and test mice were fed a high fat diet, but the test mouse diet included 0.5 mg per g-mouse per day of these mixtures. The impact of feeding combined derivatives on epididymal fat weight (EAT) and fat accumulation in mice fed a high fat diet was evaluated by comparing the test and control groups.

## Experimental

### Materials

Threonine (Thr), tryptophan (Trp), and 2-(*p*-tolyl)-ethylamine (TEA) were purchased from Sigma-Aldrich Co. (St. Louis, MO, USA). Solvents were purchased from Duksan Co. (Seoul, Korea), and other reagents were obtained from Samchun Chemicals Co. (Seoul, Korea). To feed the mice, a normal diet (ND) was supplied by BioPia Co. (Seoul, Korea) and a high-fat diet (HFD) was purchased from Dae Han Bio Link Co. (Chungcheongbuk-do, Korea).

### Microorganisms and media

*Monascus* sp. KCCM 10093 (Korea Culture Center for Microorganisms; Seoul, Korea) was used for production of orange *Monascus* pigment. Three kinds of media were used: Hiroi agar medium (w/v), which consisted of 10% sucrose, 0.5% casamino acid, 0.3% yeast extract, 0.2% NaNO_3_, 0.1% KH_2_PO_4_, 0.05% MgSO_4_·7H_2_O, 0.05% KCl, 0.001% FeSO_4_·7H_2_O, and 2% agar powder in distilled water, Mizutani medium (w/v), which consisted of 5% glucose, 2% Bacto-peptone, 0.8% KH_2_PO_4_, 0.2% CH_3_COOH, 0.1% NaCl, and 0.05% MgSO_4_·7H_2_O in distilled water, and fermentation medium (w/v) for production of orange *Monascus* pigment, which consisted of 5% glucose, 0.3% NH_4_NO_3_, 0.1% KH_2_PO_4_, 0.05% MgSO_4_·7H_2_O, 0.05% KCl, and 0.001% FeSO_4_·7H_2_O in distilled water. All of the media were adjusted to pH 6.6 prior to sterilization.

### Cultivation and orange pigment extraction

Cultivations were performed in accordance with methods presented in our previous studies.^28,30–32^ A spore suspension of *Monascus* sp. KCCM 10093 was made by adding 10 mL of sterilized distilled water to a Hiroi agar slant and shaking. For the first seed culture, the spore suspension was inoculated into 75 mL of sterilized Mizutani medium in a 500 mL baffled flask, followed by 48 h of cultivation at 30 °C. For the second seed culture, the first seed culture broth was inoculated at 7% (v/v) into 75 mL of sterilized Mizutani medium in a 500 mL baffled flask, followed by 24 h of cultivation at 30 °C. For the fermentation culture, the second seed culture broth was inoculated at 7% (v/v) into a 5 L jar fermenter containing 3 L of sterilized fermentation medium. Cultivations were performed for 168 h at 30 °C and 500 rpm with an aeration rate of 1.0 vvm.

For orange pigments extraction, as described in our previous studies,^28,30–32^ ethyl acetate was added to culture broths and incubated for 24 h in a reciprocal shaking water bath. After the ethyl acetate layer was separated using a separatory funnel, the solution was concentrated with an evaporator. During concentration, silica gel powders were added to the solution to absorb pigments. After the pigment-absorbed silica gel was mixed with 100 mL of hexane, the hexane layer containing yellow pigments was discarded. The remaining hexane was eliminated by evaporation, and then ethyl acetate was added to the concentrated silica gel. The solution was filtered to remove silica gel, and then evaporated.

### Synthesis and identification of Thr, Trp, and TEA derivatives

**Fig. 1:**
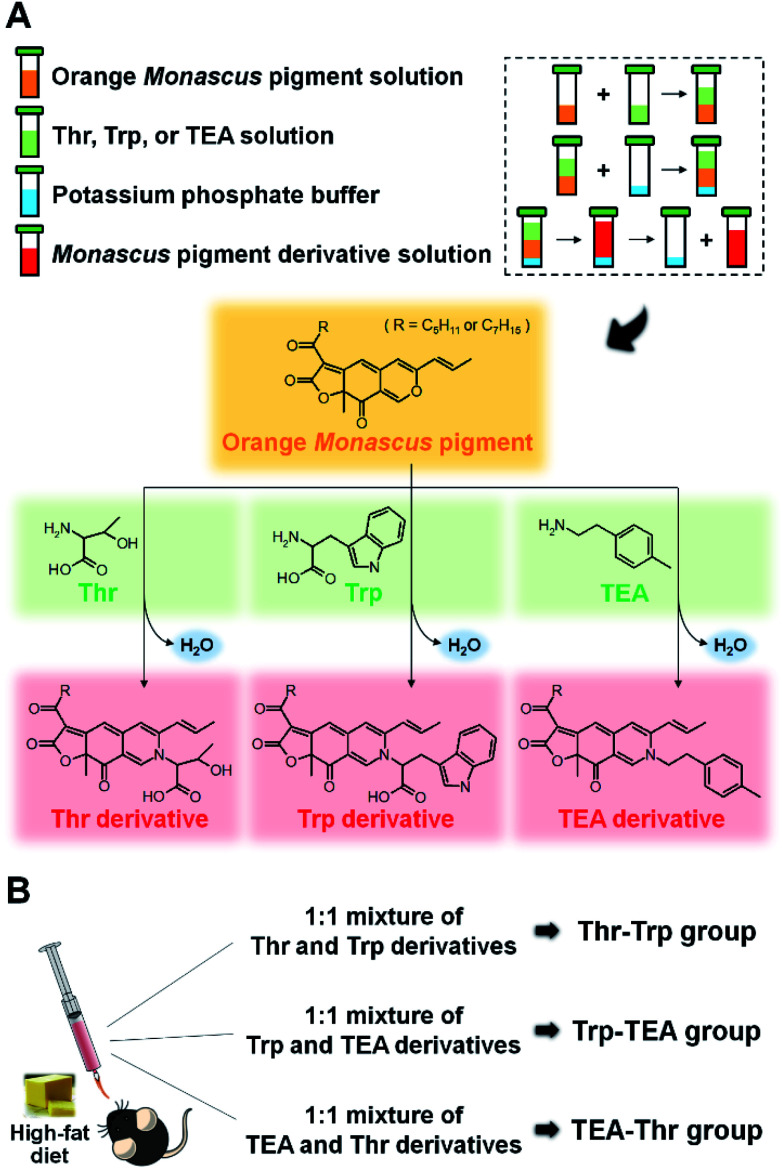
Schematic illustration of synthesis of threonine (Thr), tryptophan (Trp), and 2-(*p*-tolyl) ethylamine (TEA) derivatives from orange *Monascus* pigments (A) and mice groups divided by a combination of *Monascus* pigment derivatives (B).

The synthesis and identification of Thr, Trp, and TEA derivatives was performed in accordance with methods presented in our previous studies.^28,30–32^ The orange pigments, Thr, Trp, and TEA, were individually dissolved in ethanol at a 0.1 mM concentrations. After equal volumes of orange pigment solution and nitrogenous solution were mixed together, potassium phosphate buffer (3 M, pH 9.7) was added at 10% (v/v) to the solution. The mixture was agitated with a vortexer and incubated for 1 h at room temperature. Thr, Trp, and TEA derivatives of *Monascus* pigment were then obtained from the upper layer (Fig. 1A). For purification of Thr and Trp derivatives, the derivative solutions were spotted on TLC plates (silica gel 60 F_254_ plate; Merck, Darmstadt, Germany) and eluted with a solution of chloroform/ethanol/water (65 : 25 : 4). Red spots were recovered and dissolved in ethanol. In the case of TEA derivative, a solution of *n*-butanol/chloroform (4 : 1) was used and the major spot was scraped and dissolved in ethanol.

The synthesized derivatives were analyzed by HPLC (Acme 9000, Young-Lin Instrument; Seoul, Korea) with a GOLD C18 column (Hypersil, 250 × 4.6 mm & 5 μm), with a run time of 40 min, flow rate of 0.8 mL min^−1^, and elution gradient of distilled water/methanol from 100 : 0 to 30 : 70. A UPLC (Acquity UPLCTM, Waters; Milford, USA) equipped with an Acquity UPLC BEH C18 column (2.1 × 50 mm & 1.7 μm; Waters) was operated with a run time of 18 min, a flow rate of 0.3 mL min^−1^, and a gradient elution ratio of distilled water/MeOH (60%) to acetonitrile (40%) from 100 : 0 to 30 : 70. Pigment elution was detected by measuring absorbance at 240 nm. The molecular weights of pigment derivatives (each peak) were determined by mass spectrometry (MS) (LTQ Orbitrap XL, Thermo Scientific; Walthan, USA). The mass spectrometer was operated in ESI-positive mode, and the spray voltage was 5 kV. The capillary voltage, tube lens voltage, and capillary temperature were 35 V, 100 V, and 370 °C, respectively.

### Diets and mouse feeding tests

All animal experiments were performed in accordance with Korean Food and Drug Administration (KFDA) guidelines and were approved by the Institutional Animal Care and Use Committee (IACUC) of the Yonsei Laboratory Animal Research Center (YLARC) (permit no.: 2011-0063) in Korea. All experiments using mice were performed for 11 weeks. Laboratory rodent chow diet was used for the ND. The HFD consisted of 23.9% casein, 20.5% lard, 18.5% corn starch, 15.8% dextrose, 6% sucrose, 6% cellulose, 4.2% mineral mixture, 3% soybean oil, 1.2% vitamin mixture, 0.36% cysteine, 0.3% choline bitartrate, and 0.0017% TBHQ.

Five-week-old male C57BL/6 mice were purchased from Dae Han Bio Link Co. (Chungcheongbuk-do, Korea) and maintained under pathogen-free conditions. Fifteen mice were housed in micro-ventilated polysulfonate cages in a room kept at 23 ± 1 °C with a 12 hour light/dark cycle and 55 ± 5% relative humidity. All mice were fed with ND and tap water *ad libitum* for 1 week to stabilize their metabolic state. The mice were then divided into 5 diet groups consisting of 3 mice each. Two control groups were fed with either ND or HFD for 10 weeks. Three pigment groups (Thr–Trp, Trp–TEA, and TEA–Thr) were fed with HFD and simultaneously administered a 1 : 1 mixture of Thr and Trp derivatives, a 1 : 1 mixture of Trp and TEA derivatives, or a 1 : 1 mixture of TEA and Thr derivatives for 10 weeks by oral feeding, using a sonde, at 0.5 mg per g-mouse per day (Fig. 1B). The food intake and body weight of the mice were recorded twice per week. The reported body weight gain was determined by averaging the difference between the initial and final body weight values of each mouse.

### Analysis *via* transverse micro CT images of mouse abdomens

After 10 weeks of feeding, one mouse in each group was selected. The selected mouse fasted for 24 h before being anesthetized with avertin (2.5% tribromoethanol). Transverse micro-CT images of the mouse abdomen were accomplished with a micro-CT scanner (Skyscan-1076, Skyscan Co., Antwerp, Belgium) at a resolution of 30 μm, voltage of 100 kV, current of 100 μA, exposure of 474 ms, and a rotation step (degree) of 0.500. Micro-CT images were analyzed using CTAn software (Skyscan Co., Antwerp, Belgium). Subcutaneous and visceral fats were detected at Hounsfield units of −543.37 to +598.19.

### Weight and histology of mouse adipose tissue

After 10 weeks of feeding and a final 24 h of fasting, all mice were sacrificed. Epididymal fat tissues were excised from the mice in each group after the peritoneal cavity had been opened. Blood and foreign bodies were removed by saline solution rinse, and the weight of the adipose tissues was measured. For histological examinations, tissues were fixed with 10% neutral buffered formalin solution and embedded in paraffin. Thin sections with a thickness of 4 μm were prepared and stained using hematoxylin and eosin. The tissues were observed *via* optical microscope at a magnification of 200× (Imager Axio A1m, Carl Zeiss Co., Oberkochen, Germany).

### Statistical analysis

Data were expressed as mean ± SEM *via* analysis with SPSS software (IBM SPSS Statistics, Version 21, SPSS Inc., Chicago, IL, USA), and differences between the means were assessed by the Duncan's multiple-range test. Statistical significance was considered at *p* < 0.05.

## Results

### Preparation of *Monascus* pigment derivatives

Thr, Trp, and TEA derivatives of *Monascus* pigment were prepared according to previously described procedures.^28,30,32^ Briefly, the orange *Monascus* pigments were produced by 4 days of cultivation of *Monascus* cells in 5 L fermenters containing a modified Hiroi medium and subsequent ethyl acetate extraction. Then, Thr, Trp and TEA derivatives were synthesized from aminophilic reaction of the orange pigments with Thr, Trp and TEA, respectively (Fig. 1A). After the derivatives were purified by prep-TLC, their molecular weights were determined by LC-MS (Fig. S1†). The compounds were identified from the *m*/*z* values of 456 (R = C_5_H_11_) and 484 (R = C_7_H_15_) for Thr derivative, 472 (R = C_5_H_11_) and 500 (R = C_7_H_15_) for Trp derivative, and 541 (R = C_5_H_11_) and 569 (R = C_7_H_15_) for TEA derivative, respectively.

### The effect of *Monascus* pigment derivatives on the weight gain of mice

In order to assess whether the supplementation of combined Thr, Trp and TEA derivatives on mice growth is effective against obesity and to determine the best combination, three 1 : 1 derivative mixtures (Thr–Trp, Trp–TEA, and TEA–Thr groups) were fed to mice for 10 weeks. The overall diet consumption rate and weight gain for each group were analyzed and are summarized in Table 1. The diet consumption rates of the ND (normal diet) and HFD (high fat diet) groups were 2.27 and 2.28 g day^−1^, respectively. The diet consumption rates of three pigment groups were 2.34–2.66 g day^−1^. There were no statistically significant differences in consumption rates between any of the groups, as we expected. Despite the similar diet consumption rates, there were relatively significant differences in their weight gains. The highest weight gain (12.04 g per mouse) was observed in the HFD group, which was an increase of 11.6% compared to the results from the ND group (10.79 g per mouse). However, relatively lower weight gain was achieved in the pigment derivative groups, in the range of 7.48–9.59 g per mouse, decreasing by 20.3–37.9% body weight compared to the HFD group. The supplement of Thr–Trp derivatives resulted in the lowest weight increase (7.48 g per mouse), which was 37.9% lower than the HFD group.

**Table 1:** The effect of *Monascus* pigment derivatives on the body weight gain and diet consumption rate in mice^a^

Group	Body weight (g per mouse)		Weight gain (g per mouse)	Diet consumption rate (g per mouse per day)
	Initial	Final		
ND	18.53 ± 0.45^a^	29.32 ± 0.28^a^	10.79 ± 0.70^ab^	2.27 ± 0.77^a^
HFD	18.33 ± 0.21^a^	30.37 ± 2.48^a^	12.04 ± 2.29^a^	2.28 ± 0.24^a^
Thr–Trp	16.97 ± 0.45^b^	24.45 ± 0.30^b^	7.48 ± 0.75^c^	2.34 ± 0.31^a^
Trp–TEA	16.93 ± 0.42^b^	25.66 ± 2.11^b^	8.72 ± 1.69^bc^	2.43 ± 0.34^a^
TEA–Thr	16.77 ± 0.60^b^	26.36 ± 1.05^b^	9.59 ± 1.66^abc^	2.66 ± 0.84^a^

a Each value is a mean ± SEM (*n* = 3). Different superscript letters in each column indicate that values are significantly different at *p* < 0.05 (Duncan's test). ND = mice group fed a normal diet without *Monascus* pigment derivatives; HFD = mice group fed a high-fat diet without *Monascus* pigment derivatives; Thr–Trp = mice groups fed a high-fat diet with Thr and Trp derivatives of *Monascus* pigment; Trp–TEA = mice groups fed a high-fat diet with Trp and TEA derivatives of *Monascus* pigment; TEA–Thr = mice groups fed a high-fat diet with TEA and Thr derivatives of *Monascus* pigment.

### The effect of *Monascus* pigment derivatives on lipid reduction in mice

**Fig. 2:**
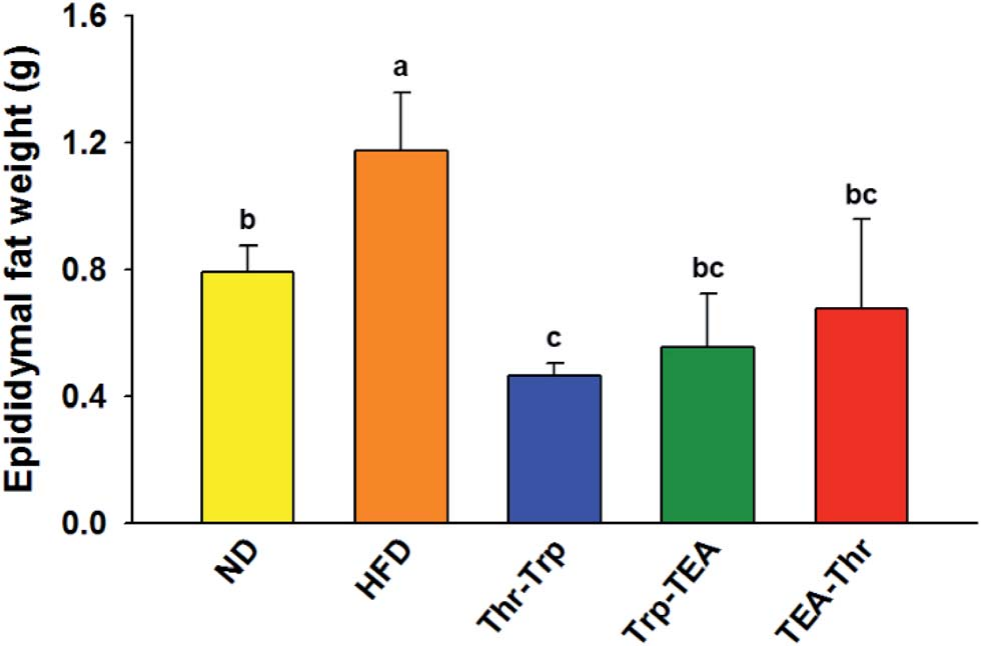
Epididymal fat weight of a C57BL/6 mouse fed with each test diet. Values are the mean ± SEM (*n* = 3). Different letters on rectangles indicate that the values are significantly different at *p* < 0.05 (Duncan's test). ND = mice group fed a normal diet without *Monascus* pigment derivatives; HFD = mice group fed a high-fat diet without *Monascus* pigment derivatives; Thr–Trp = mice groups fed a high-fat diet with Thr and Trp derivatives of *Monascus* pigment; Trp–TEA = mice groups fed a high-fat diet with Trp and TEA derivatives of *Monascus* pigment; TEA–Thr = mice groups fed a high-fat diet with TEA and Thr derivatives of *Monascus* pigment.

**Fig. 3:**
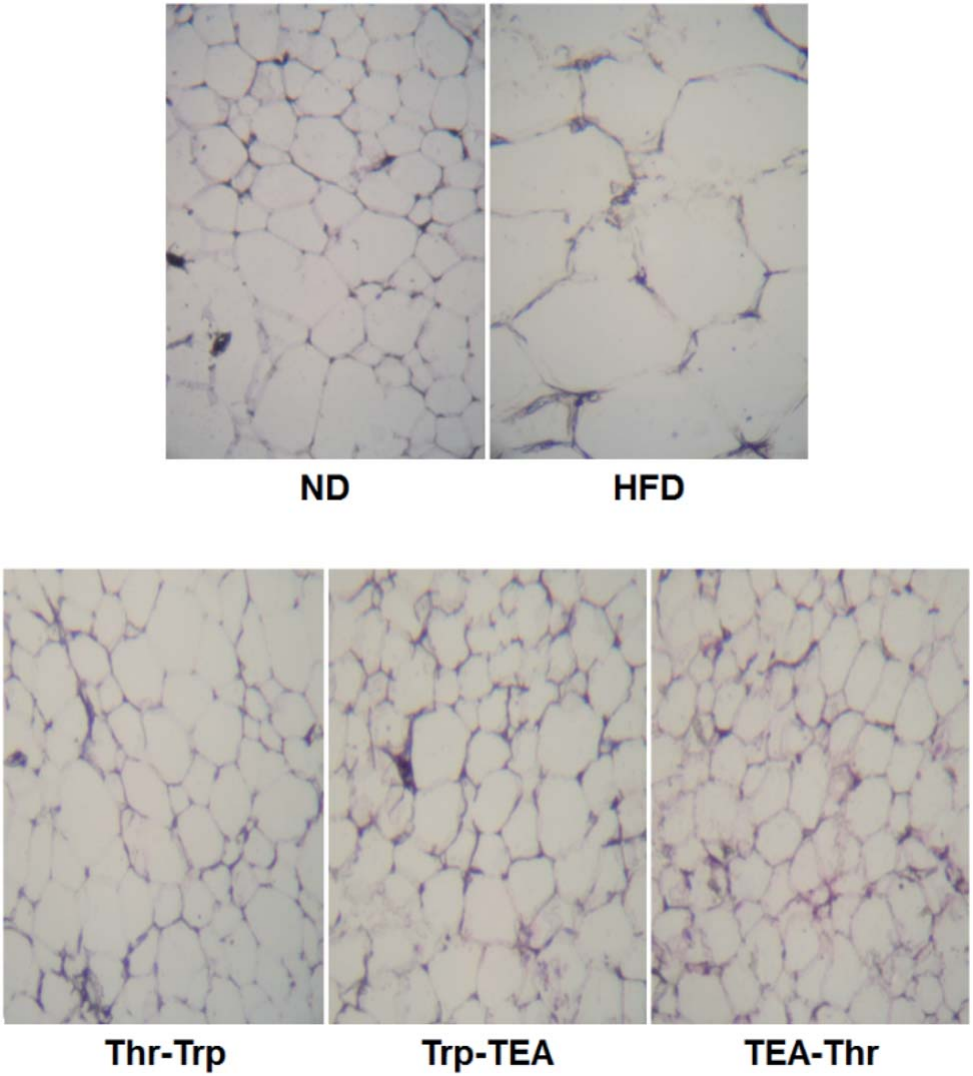
Epididymal fat photomicrographs (magnification, ×200) of a C57BL/6 mouse fed with test diets. ND = mice group fed a normal diet without *Monascus* pigment derivatives; HFD = mice group fed a high-fat diet without *Monascus* pigment derivatives; Thr–Trp = mice groups fed a high-fat diet with Thr and Trp derivatives of *Monascus* pigment; Trp–TEA = mice groups fed a high-fat diet with Trp and TEA derivatives of *Monascus* pigment; TEA–Thr = mice groups fed a high-fat diet with TEA and Thr derivatives of *Monascus* pigment.

To evaluate the effect of *Monascus* pigment derivatives on lipid reduction in mice, the amounts and sizes of epididymal adipose tissue (EAT) were investigated. The EAT weights of mice in each group are presented in Fig. 2. While the EAT weight of the HFD group (1.17 g per mouse) was 1.5 times higher than that of the ND group (0.79 g per mouse), when the combined *Monascus* pigment derivatives were administered to mice, EAT weights were all reduced compared to the HFD group. The EAT weights of the three pigment groups were 0.46–0.68 g, or 41.9–60.7%, lower than the HFD group. Among the five groups, the lowest EAT weight was observed in the Thr–Trp treatment group (60.7%) or 0.46 g per mouse EAT, which showed similar anti-obesity effects to the results of the weight gains with the Thr–Trp derivatives group. Besides affecting the EAT weights in mice, *Monascus* pigment derivatives also affected the morphological appearance and size changes of the EAT at the microscopic level. Fig. 3 displays EAT photomicrographs of C57BL/6 mice fed with test diets at 200× magnification. The HFD group visually had the largest EAT size, suggesting there was more cell differentiation and accumulation of lipid in the cell. The EAT sizes of mice in the three pigment group were visually smaller than those in the HFD group, and there was little to no difference compared to the ND group.

**Fig. 4:**
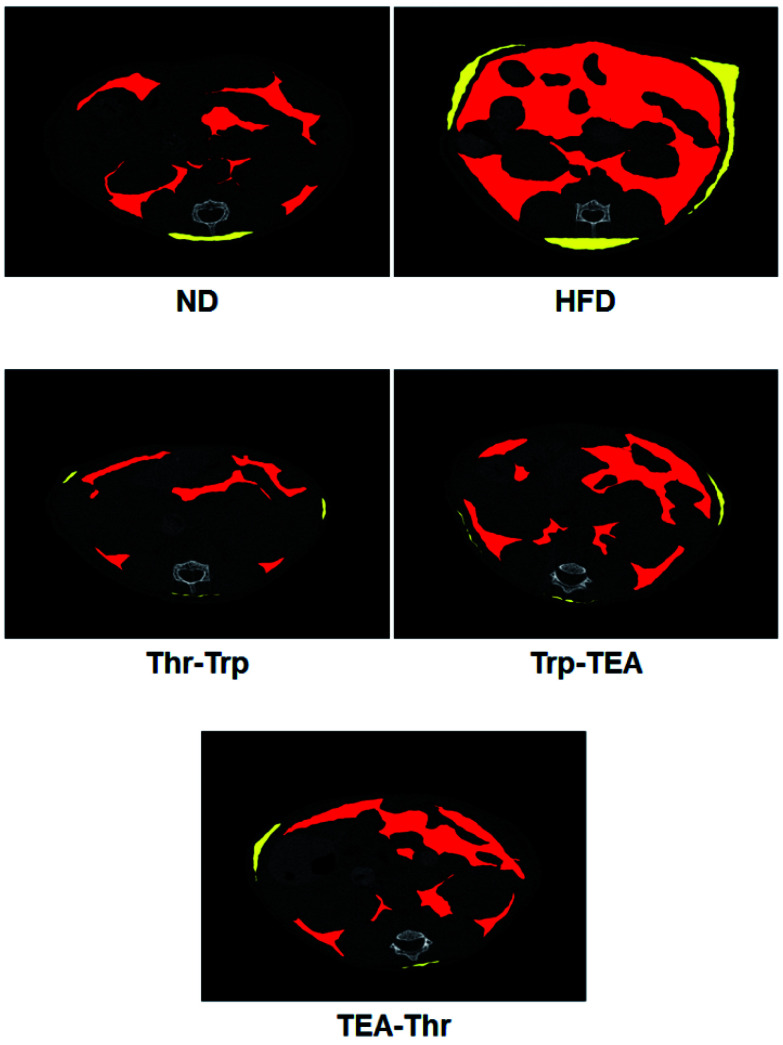
Transverse micro CT images of C57BL/6 mice abdomens which have been fed test diets. Red and yellow colors are subcutaneous and visceral fats, respectively. ND = mice group fed a normal diet without *Monascus* pigment derivatives; HFD = mice group fed a high-fat diet without *Monascus* pigment derivatives; Thr–Trp = mice groups fed a high-fat diet with Thr and Trp derivatives of *Monascus* pigment; Trp–TEA = mice groups fed a high-fat diet with Trp and TEA derivatives of *Monascus* pigment; TEA–Thr = mice groups fed a high-fat diet with TEA and Thr derivatives of *Monascus* pigment.

Further investigation for overall lipid reduction was performed using transverse micro-CT images of the mice abdomens. Red and yellow colors indicate subcutaneous and visceral fats, respectively (Fig. 4). The results are in agreement with previous results of weight gains and lipid reductions: the subcutaneous and visceral fat layers in the HFD group were thicker than those in the ND group (HFD *vs.* ND in Fig. 4). Compared to the HFD group, the subcutaneous fat layers of three pigment groups were significantly thinned and few visceral fats were observed in the pigment derivatives groups.

## Discussion

In general, anti-obesity effects are closely related to one of four different mechanisms.^33^ The first mechanism is to supress the appetite at the central nervous system level. By reducing the sensation of hunger and increasing the sensation of satiety, less food is consumed, resulting in anti-obesity effects. The second mechanism is to interfere with nutrient absorption. The inhibition of digestive enzymes such as lipase and amylase hinders the digestion of foods, which suppresses the intestinal absorption of dietary fats and sugars. The third mechanism is to increase energy expenditure (thermogenesis). The activation of the uncoupling protein (UCP1–3) in the mitochondria elicits anti-obesity effects by enhancing thermogenesis that converts the energy obtained from foods to heat rather than fat. The last mechanism is to decrease adipogenesis. The inhibition of the proliferation and differentiation of adipocyte precursors reduces hyperplasia and hypertrophy of adipocytes, leading to a decrease in fat accumulation.

Our previous work confirmed that Trp derivative is related to interference of nutrient absorption. In particular, the Trp derivative inhibited porcine pancreatic lipase (digestive enzyme), with an IC_50_ of 61.2 μM.^25^ The Trp derivative functioned as a non-competitive inhibitor against lipase, and its inhibition was accomplished by binding with allosteric sites on the enzyme. Lipase inhibition by the Trp derivative depressed the hydrolytic conversion of fats into generic molecules of glycerol and fatty acid, which reduced fat absorption in the intestine.^25,27^ When the Trp derivative was fed to mice at 0.1 or 0.2 mg per g-mouse per day for 6 weeks, intraperitoneal fat weights were reduced up to 27.8% compared to the HFD group.^27^

On the other hand, the TEA derivative was shown to be capable of decreasing adipogenesis. When the TEA derivative was tested in 3T3-L1 cells at a concentration of 10 μM, the expressions of the adipogenic transcriptional factors of PPARγ and C/EBPα were decreased by 80% and 70%, respectively.^28^ Inhibition by the TEA derivative suppresses cell differentiation from pre-adipocytes into mature fat cells, which subsequently decreases fat storage in adipose tissue.^28^ When the TEA derivative was fed to mice at 0.1, 0.2 or 0.4 mg per g-mouse per day for 17 weeks, epididymal fat weights were reduced up to 56.0% compared to the HFD group.^28^

These results have experimentally proven that both Trp and TEA derivatives have anti-obesity activity as well as play a key role as inhibitors of absorption and storage of fats. In light of these facts, we hypothesized that the combined treatment of Trp and TEA derivatives, with their different inhibitory mechanisms, would improve anti-obesity effects *vs.* discrete usage. To test our hypothesis, combined Trp–TEA derivative treatments were examined and compared to other derivative combinations.

In contrast to our expectations, anti-obesity effects were attained in the order of the Thr–Trp group > the Trp–TEA group > the TEA–Thr group (Table 1 and Fig. 2). The Thr–Trp group exhibited higher anti-obesity effects than the Trp–TEA group, suggesting the combination of Thr with Trp derivatives is more effective than those with TEA derivatives. This is probably due to the presence of the Thr derivative, which improves efficacy when it is combined with another pigment derivative. Our previous studies raised the potential that the Thr derivative could hamper HMG-CoA reductase,^26^ which is a key enzyme converting HMG-CoA to mevalonate, a rate limiting step in cholesterol biosynthesis.^34,35^ The supplementation of the Thr derivative inhibited HMG-CoA reductase by up to 38%, and subsequently the cholesterol level was significantly decreased.^26^ Further dietary effects with mice showed that mice fed 2% (w/w) cholesterol plus diet medium increased weight by up to 51% while the test with Thr derivative treatment at 0.02% (g per g mouse weight) in similar experimental conditions showed little to no impact on weight increase.^22,26^ Furthermore, the Thr derivative also has an inhibitory activity on cholesteryl ester transfer protein (CETP), which is an enzyme responsible for moving triglycerides and cholesterol esters between very low-density lipoprotein (VLDL), low-density lipoprotein (LDL), and high-density lipoprotein (HDL).^30,36^ The inhibition of CETP not only decreases LDL cholesterol levels but also increases the high-density lipoprotein (HDL) level.^36^ Jang, *et al.*^30^ highlighted that the Thr derivative treatment at 2 μM concentration decreased the CETP activity by 45% and its IC_50_ value was about 1.0 μM, suggesting further inhibition effects at low dosage when it is combined with other pigment derivatives. Similarly, our previous work noted that when mice were fed the high-cholesterol diet (HCD) supplemented with the Thr derivative at 0.1 or 0.2 mg per g-mouse per day for 10 weeks, both the total and LDL levels were reduced and weight gain was also decreased up to 17.3% compared to the HCD group.^26^ Considering these beneficial effects of Thr derivative consumption on dietary, cholesterol, and CETP inhibition, the Thr derivative sufficiently contributes to increase the anti-obesity effect when it is combined with the Trp derivative.

Although the Thr derivative would affect reductions in both body weight and fat content, it should not be overlooked that a combination of the Trp derivative considerably contributed to high anti-obesity effects of the Thr–Trp group. A large portion of the anti-obesity effects of the Thr–Trp group is probably caused by the lipase inhibitory activity of the Trp derivative. Actually, lipase inhibition is one of the main targets for obtaining high anti-obesity effects. For instance, it is known that commercial anti-obesity drugs such as orlistat and sibutramine inhibit lipase reactions.^32^ Therefore, we conclude that the combination of Trp and Thr derivatives, with their different anti-obesity activities, provide synergic effects on mouse weight gain, EAT cell growth, and lipid accumulation in the cells.

As aforementioned, the combined Thr–Trp treatment resulted in considerable fat loss effects (60.7%), which were greater than those observed in the Trp–TEA group (53.0%), the Trp-only group (27.8%), and the TEA-only group (56.0%) (Fig. 2).^27,28^ In contrast to the Thr–Trp group, the lowest fat loss (41.9%) was observed in the TEA–Thr group compared to the Thr–Trp (60.7%) and the Trp–TEA (53.0%) groups, mostly due to the lack of lipase inhibition and the short experimental period. Adipogenic cell differentiation for fat storage proceeds between pre-adipocytes and mature fat cells, which takes a relatively longer reaction time. To assess the anti-obesity effects of the TEA derivative on adipogenic cell differentiation leading to fat storage in adipose tissue, an extended experiment time of 17 weeks was executed.^28^ In the case of lipase inhibition (Trp derivative) for fat absorption, however, a short test period of 6 weeks was sufficient to observe fat loss effects on mice.^27^ The current work with combinations of *Monascus* pigment derivatives was carried out for 10 weeks, thus suggesting the TEA derivative (adipogenic differentiation inhibitor) achieved lower anti-obesity activity and consequently resulted in the lowest fat loss in the TEA–Thr group.

In summary, these results experimentally prove the inhibitory effects of combining both *Monascus* pigment derivatives for anti-obesity activity. Each derivative compound has a different inhibitory pathway, and the combination of the two significantly hampers mouse weight gain and lipid storage in the abdomen and decreases the size of EAT.

## Conclusions

The combination of Thr and Trp *Monascus* pigment derivatives showed the highest anti-obesity effect in mice. The anti-obesity effects were obtained in the order of the Thr–Trp group > the Trp–TEA group > the TEA–Thr group. The anti-obesity effects were partially confirmed by the comparison of body weights and EAT weights and were photographically confirmed by the decreased EAT size and decreased abdominal thickness. These combined derivatives with their anti-obesity effects will provide potential research avenues for bio-functional and pharmaceutical applications.

## Conflicts of interest

There are no conflicts to declare.

## Supplementary Material

RA-010-C9RA08036H-s001
