# Supplementary material for: In vivo evaluation of the anti-obesity effects of combinations of Monascus pigment derivatives
Source: RSC Adv. 2020 Jan 8;10(3):1456–62. doi: 10.1039/c9ra08036h (PMC9047284; doi:10.1039/c9ra08036h)
Supplement: RA-010-C9RA08036H-s001 [file RA-010-C9RA08036H-s001.pdf]

Electronic Supplementary Material (ESI) for RSC Advances.
This journal is © The Royal Society of Chemistry 2019

Supplementary Information

In vivo evaluation of the anti-obesity effects of combinations of *Monascus* pigment derivatives

Deokyeong Choe,[‡a] Hyun Ho Jung,[‡b] Daehwan Kim,[c] Chul Soo Shin,[b] Tony Vaughn Johnstona and Seockmo Ku*[a]

[a]*Fermentation Science Program, School of Agriculture, College of Basic and Applied Sciences, Middle Tennessee State University, Murfreesboro, TN 37132, USA*

[b]*Department of Biotechnology, College of Life Science and Biotechnology, Yonsei University, Seoul 03722, South Korea*

[c]*Department of Biology, Hood College, Frederick, MD 21701, USA*

* Corresponding author: seockmo.ku@mtsu.edu

‡ These authors contributed equally.

A

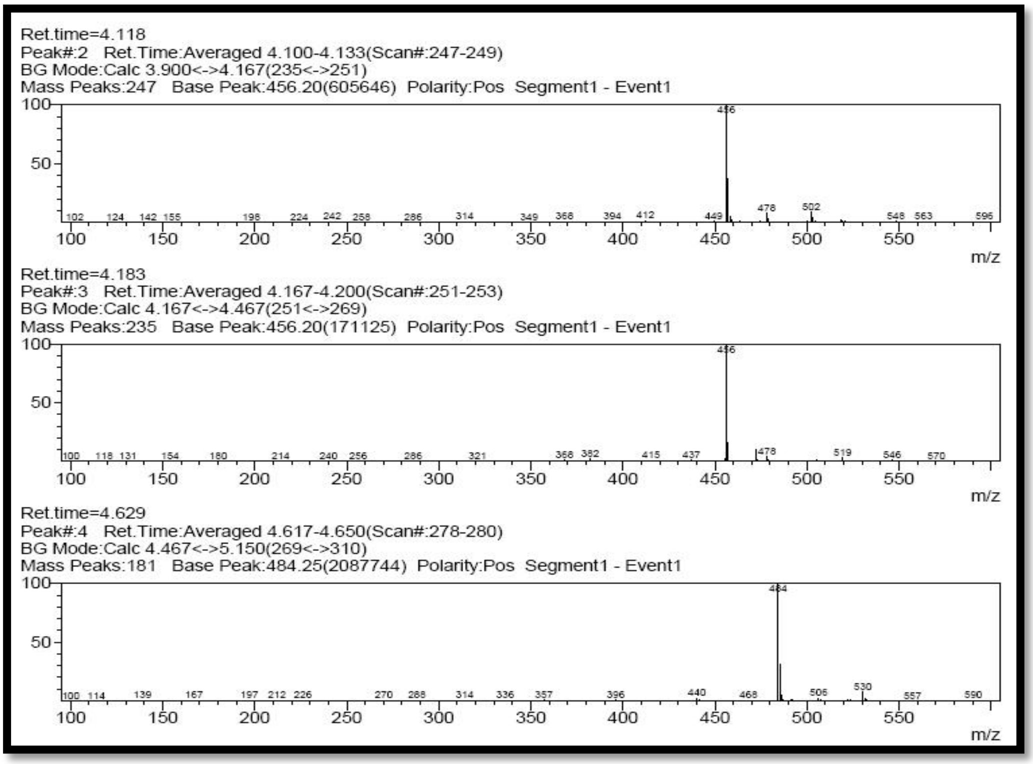
Ret.time=4.118
Peak#:2 Ret.Time:Averaged 4.100-4.133(Scan#:247-249)
BG Mode:Calc 3.900<->4.167(235<->251)
Mass Peaks:247 Base Peak:456.20(605646) Polarity:Pos Segment1 - Event1
m/z
Ret.time=4.183
Peak#:3 Ret.Time:Averaged 4.167-4.200(Scan#:251-253)
BG Mode:Calc 4.167<->4.467(251<->269)
Mass Peaks:235 Base Peak:456.20(171125) Polarity:Pos Segment1 - Event1
m/z
Ret.time=4.629
Peak#:4 Ret.Time:Averaged 4.617-4.650(Scan#:278-280)
BG Mode:Calc 4.467<->5.150(269<->310)
Mass Peaks:181 Base Peak:484.25(2087744) Polarity:Pos Segment1 - Event1
m/z

B

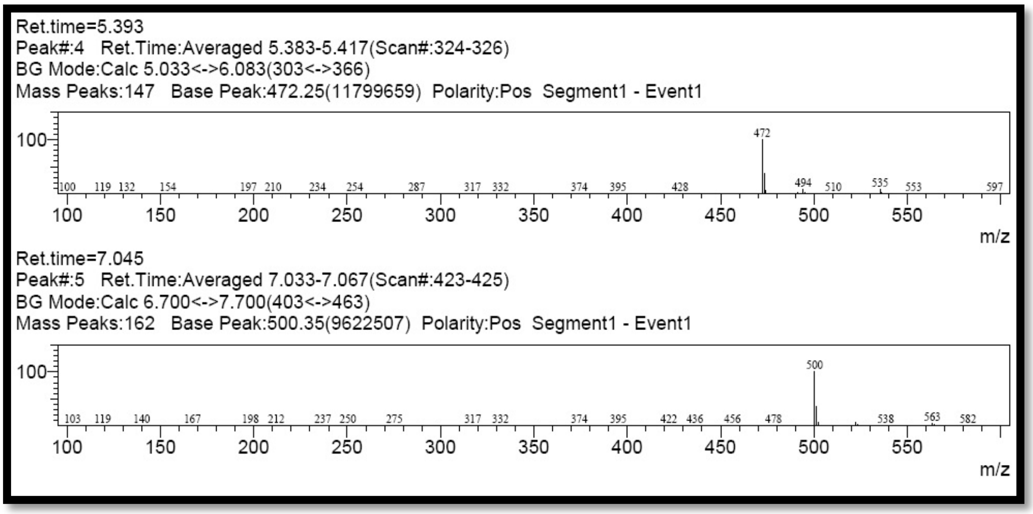
Ret.time=5.393
Peak#:4 Ret.Time:Averaged 5.383-5.417(Scan#:324-326)
BG Mode:Calc 5.033<->6.083(303<->366)
Mass Peaks:147 Base Peak:472.25(11799659) Polarity:Pos Segment1 - Event1
m/z
Ret.time=7.045
Peak#:5 Ret.Time:Averaged 7.033-7.067(Scan#:423-425)
BG Mode:Calc 6.700<->7.700(403<->463)
Mass Peaks:162 Base Peak:500.35(9622507) Polarity:Pos Segment1 - Event1
m/z

C

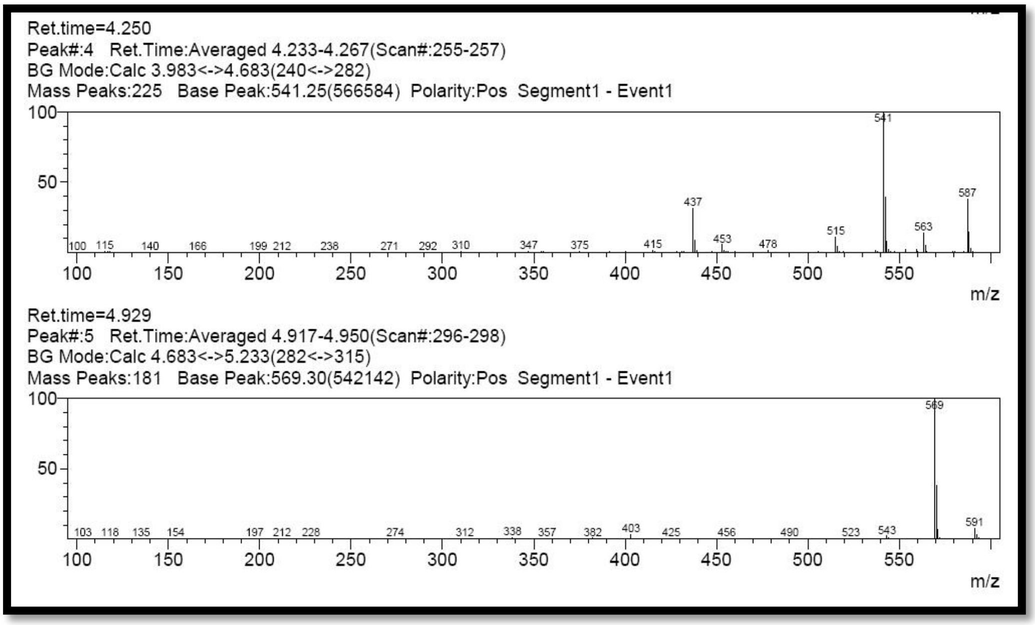

Figure S1. LC-MS spectrometric analysis of *Monascus* pigment derivatives.

A: Thr derivative; B: Trp derivative; C: TEA derivative.
